# A Mixed-Methods Approach to Evaluating the Internal Validity of the Reactive Strength Index

**DOI:** 10.3390/sports7070157

**Published:** 2019-06-27

**Authors:** Talin Louder, Brennan J. Thompson, Nile Banks, Eadric Bressel

**Affiliations:** 1 Division of Kinesiology and Sport Management, University of South Dakota, Vermillion, SD 57069, USA; 2 Department of Kinesiology and Health Science, Utah State University, Logan, UT 84321, USA; 3 School of Kinesiology, Applied Health, and Recreation, Oklahoma State University, Stillwater, OK 74078, USA; 4 Sport Performance Research Institute, Auckland University of Technology, Auckland 0627, New Zealand

**Keywords:** RSI, depth jump, plyometric

## Abstract

The reactive capacity of the muscle-tendon complex is commonly assessed using the reactive strength index (RSI). Conventionally, the RSI is a ratio of rebound jump height to ground contact time in depth jumping. Several assumptions regarding the linear mechanics acting through the whole-body center of gravity may threaten the internal validity of computation and interpretation of RSI scores. First, it is common for rebound jump height to be predicted from rebound jump flight time. This assumes that the angular positioning of body segments is equivalent at the time instances of rebound jump take-off and landing. Prior literature supports a mixed-methods approach for computing the RSI that is void of this assumption. The mixed-methods approach gives a more valid estimation of rebound jump height. In this approach, rebound jump height is estimated from rebound jump take-off velocity of the whole-body center of mass. This is accomplished by subtracting an estimate of impact velocity, acquired using videography, from change in whole-body center of mass velocity estimated from integrated vertical ground reaction force data. Second, it is often assumed that vertical displacement of the whole-body center of mass during the drop phase of the depth jump is predicted perfectly from the height of the platform used to perform the drop. This assumption may affect the internal validity of comparing RSI scores across individuals and within individuals performing depth jumps from varied heights. The purpose of the present study was to investigate the internal validity of RSI scores computed using the conventional approach and impact velocity variability, which may affect the interpretation of RSI scores. Seventy physically active young adults performed depth jumps from drop heights of 0.51, 0.66, and 0.81 m. RSI was computed using the conventional approach and a mixed-methods approach featuring the use of 2-dimensional videography, body segment parameters, and force platform dynamometry. The two computational methods were compared using linear regression performed on data from each drop height. In addition, a 2 (computational method) by 3 (drop height) Analysis of Variance (ANOVA) was performed to evaluate for main effects and interactions in RSI data. Multiple one sample *t*-tests were performed to compare estimated and theoretical impact velocities. The ANOVA revealed no main effect or interactions between computational approaches (*p* = 0.467–0.938). Linear regression revealed moderately strong associations between RSI scores computed using the conventional and mixed-methods approaches (*R*^2^ = 0.685–0.741). Moreover, linear regressions revealed that the conventional approach tends to overestimate the mixed methods approach for RSI scores below 1.0 and underestimate the mixed methods approach for RSI scores above 1.0. Lastly, estimated impact velocities were observed to be as much as 13% lower versus theoretical (*p* < 0.001). Researchers with access to motion capture and force platform technology may consider using a mixed-methods approach for computing the RSI, which likely maximizes the internal validity of scores. In addition, results suggest for practitioners to practice caution when comparing conventional RSI scores across individuals.

## 1. Introduction

Actuation of tension within the muscle-tendon complex is realized from integration of active and passive elements. Hill-type models of muscle tension generally comprise an active contractile element and passive elastic elements arranged serially and in parallel [1,2,3]. Moreover, the mechanical behavior of the muscle-tendon complex is typically categorized into one of four principal actions: concentric, isometric, eccentric, and reactive [4]. Humans’ propensity for producing muscular strength and power during concentric, isometric, and eccentric actions is effectually described by the force-length and force-velocity dependencies of active and passive muscle-tendon elements [1,2,3]. Modelling the reactive capacity or strength of the muscle-tendon complex is more contemporary and has attracted considerable attention in recent literature.

Reactive strength was formally introduced in 1995 by Warren Young [5] as a measure of ability for changing quickly from eccentric to concentric muscle-tendon action during countermovement and depth jumping. Intuitively, reactive strength is also suggested to reflect one’s capacity for distributing external loads through the muscle-tendon complex [6]. Reactive strength is dependent on feedforward motor control processes and supported by time-sensitive spinal reflexivity [7]. Development of active tension in anticipation of external loading reflects the importance of predicting foot-to-ground impact momentums [7]. Moreover, it is vital that feedforward anticipation be scaled according to ground reaction force magnitude, timing, and direction [7]. For instance, it is suggested that excessive active tension developed in anticipation of external loading may result in harmful levels of stress applied through the muscle-tendon complex [7]. Conversely, insufficient active tension developed in anticipation of external loading may alter joint decelerations [7] and partition load through supporting structures including ligaments, joints, and bones.

Accurate feedforward anticipation of external forces facilitates loading through the passive elements of the muscle-tendon complex and prepares key time-sensitive spinal reflex feedback mechanisms [7]. When landing from a jump, vertical ground reaction forces are observed to peak approximately 100 ms following foot-to-ground impact [8]. Given that voluntary activation of muscle involves a latent period of 100–200 ms [9], ground reaction forces sustained during jump landing impacts are effectively processed through feedforward anticipation and spinal reflexivity (30–60 millisecond latency) [9]. Thus, reactive strength is supported by the time-sensitive myotatic reflex and infers one’s ability to safely distribute stress from external loading across the active and passive components of the muscle-tendon complex [6]. Conversely, reactive strength is tempered by the inverse myotatic reflex, which promotes relaxation of stress within the muscle-tendon complex [6]. 

For jumps that require anticipation of ground reaction forces (e.g., the depth jump), reactive strength is typically estimated using the reactive strength index (RSI) [6]. Inherently, measures of strength are kinetic-based. The RSI is not kinetic-based, rather it is computed as a spatiotemporal ratio of rebound jump height to ground contact time. While the RSI does permit inference into the linear kinetics of the whole-body center of mass, it includes the following assumptions:

1. **Assumption #1:** Rebound jump height is often estimated by inputting flight time to an equation of constant acceleration. This approach assumes that the whole-body center of mass is the same distance above the ground at the time instances of rebound jump take-off and rebound jump landing. Specific to the lower extremity, it is typical for the angular positioning of body segments to be more flexed at jump landing versus jump take-off. Therefore, this assumption may threaten the internal validity of conventional RSI computation.
2. **Assumption #2:** The RSI assumes that vertical displacement of the whole-body center of mass during the drop phase of the depth jump is mathematically equivalent to the height of the platform used to perform the drop. This assumption does not introduce threats to internal validity in the conventional computation of the RSI. However, this assumption may introduce threats to internal validity specific to the interpretation of RSI scores. Often, RSI scores are interpreted within the context of drop height. This interpretation assumes that for any two depth jumps, the impact velocity sustained when landing from the drop phase of the depth jump does not vary and can be predicted perfectly using the height of the platform. However, variability in technique when stepping off the platform likely leads to variability in impact velocity that deters from this theoretical assumption.

Assumption #1 has been observed in prior literature to produce measurement error when estimating rebound jump height in depth jumping [10]. To maximize internal validity, a mixed-methods approach may be used to estimate rebound jump height that combines videography, body segment parameters, and force platform dynamometry [10]. This approach provides an estimate of rebound jump take-off velocity by taking the difference between estimated impact velocity acquired via videography and change in velocity of the whole-body center of mass acquired via integrated force platform data corresponding to ground contact time. Taking the difference of these two velocity values gives an estimate of rebound jump take-off velocity that is then inputted into equations of constant acceleration to estimate rebound jump height. In prior literature [10], the mixed-methods approach has been validated to estimate rebound jump height to an accuracy of 7 ± 13 mm. In this same investigation, the conventional flight time method produced as much as 8.4% of measurement error specific to estimating rebound jump height. Therefore, using a mixed-methods approach, the purpose of the present study was to investigate the internal validity of RSI scores computed using the conventional approach and impact velocity variability, which may affect the interpretation of RSI scores. This is timely, considering that the RSI is an increasingly utilized outcome measure in sport and clinical movement research.

## 2. Materials and Methods

### 2.1. Participants

Seventy physically active young adults (22.1 ± 1.9 years; 77.8 ± 15.5 kg; 176.2 ± 12.4 cm; male = 36; female = 34) with no recent history of lower extremity injury volunteered to participate in this study. Participants, recruited from the local community, were approved for inclusion if they were between the ages of 18 and 30 years and confirmed no recent history (3 months) of lower extremity injury. Participants, on average, self-reported that they engaged in moderate to vigorous intensity exercise 4.2 ± 0.7 days per week. Prior to study involvement, participants read and provided consent through signature on an informed consent document that was reviewed and approved by the University Institutional Review Board (6966).

### 2.2. Procedures

Participants attended a single data collection lasting approximately 1 h. Sessions were conducted in the morning, with no controls placed on the immediate history of diet, sleep patterns, and other physiological variables that could have been confounders to the investigation. Upon arrival to the laboratory, participants were instructed in a familiarization session. The familiarization session lasted approximately 5–10 min, allowed participants to practice the depth jumping technique, and permitted members of the research group to instruct and observe technique prior to data acquisition. Data acquisition commenced after a rest period of 20 min post-familiarization. During the rest period, markers were affixed according to the 14-segment model proposed by de Leva [11]. Data acquisition and familiarization were performed on the same day, while adhering to rest and jump volume recommendations provided by the National Strength and Conditioning Association [12]. 

The experimental protocol required participants to perform three repetitions of depth jumping at drop heights of 0.51, 0.66, and 0.81 m. For each trial of depth jumping, participants were instructed to initiate the drop phase by “stepping forward off the box with their preferred foot.” Participants were then instructed to “land from the drop with both feet impacting the ground simultaneously” and then “perform a maximal jump upwards following impact with the ground and focus on jumping as high and as quickly as possible.” A member of the research group monitored each trial visually to ensure that both feet impacted fully onto a force platform that was recessed to be level with the laboratory floor. Any trial where the feet did not impact the force platform fully was declared unsuccessful and then repeated. 

### 2.3. Data Analysis

#### 2.3.1. Videography and Body Segment Parameters

For each trial of depth jumping, video data were captured using a high-speed camera (Model EX-F1, Casio, Shibuya, Tokyo, Japan) aligned perpendicular to the sagittal plane of motion and placed at a distance of 5 m from the participant. The camera was levelled and secured at a height of 0.67 m above the laboratory floor. Video data were sampled at 300 Hz.

Using video data, de Leva [11] segment endpoint positions were digitized using Kinovea (0.8.27) open-source software. Endpoint locations were digitized from initiation of movement through full foot contact with the force platform following the drop phase. Full foot contact was determined as the point in time where ankle motion transitioned from dorsiflexion to plantarflexion. For each trial, full foot contact was evaluated subjectively by visual inspection of video data performed by a single member of the research group. Using this convention for the endpoint of videography analysis padded data to ensure accuracy in estimating impact velocity of the WBCoM. Since impact velocity is achieved at the time instance where the feet impact the ground, including data points beyond impact ensured that impact velocity was not aliased in digitization. Post-digitization, endpoint positions were imported into a custom Microsoft Excel (Microsoft Corporation, Redmond, WA, USA) template and processed through a low-pass, recursive, 4th order Butterworth filter (6 Hz cut-off frequency determined via residual analysis) [13]. Additionally, in Microsoft Excel (Microsoft Corporation, Redmond, WA, USA), vertical whole-body center of mass (WBCoM) position was modelled using filtered segment endpoint data and weighting tables provided by de Leva [11]. Estimating WBCoM position using videography and de Leva [12] body segment parameters is observed to have acceptable validity when compared against doubly integrated ground reaction force data [10,14].

WBCoM vertical velocity at every time point between initiation of movement and full foot contact with the force platform following the drop phase was estimated using the first central difference method [15]. Estimated impact velocity was calculated by selecting the maximal value for vertical WBCoM velocity. Theoretical impact velocities (m*s^−1^) for each of the three drop heights (m) were computed by inputting drop height into Equation (1).

(1) $$Theoretical\;Impact\;Velocity=\sqrt{19.62\times Drop\;Height}.$$

#### 2.3.2. Force Platform Dynamometry

For each trial of depth jumping, vertical ground reaction force (GRF) data were captured using a tri-axial force platform (Model FP4080, Bertec Corporation, Columbus, OH, USA) that was recessed to be flush with the laboratory floor. GRF data were sampled at 1000 Hz. Acquisition of GRF data was set for 20 s per trial but was initiated and terminated manually once each depth jump movement had been captured in full.

**Figure 1:**
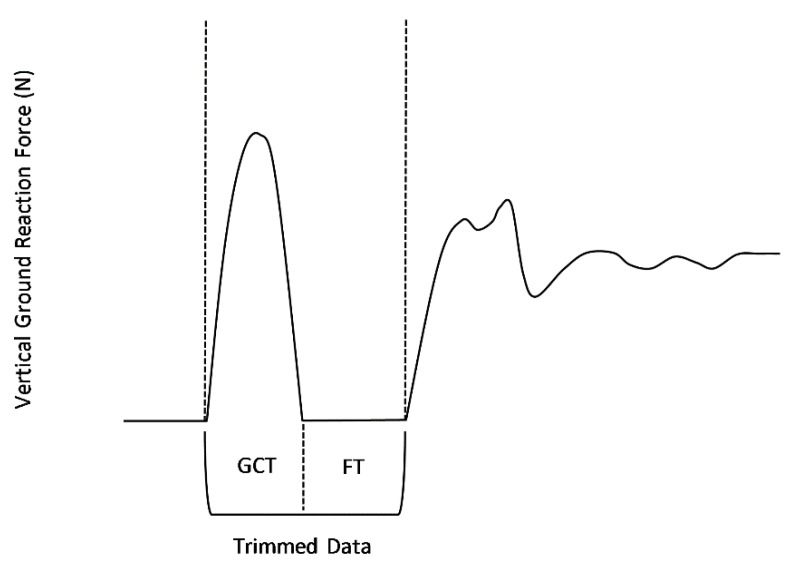
Exemplar representation of time-series vertical ground reaction force data acquired during a single trial of depth jumping. Trimmed data were used to estimate ground contact time (GCT) and flight time (FT).

GRF data were imported into Microsoft Excel (Microsoft Corporation, Redmond, WA, USA) and post-processed. GRF data were first processed through a low-pass, recursive, 4th order Butterworth filter (100 Hz cut-off frequency). Using methods described previously [16], GRF data were then trimmed to begin at the time instant of ground impact following the drop phase and end at the time instant of rebound jump landing (Figure 1). Using GRF data, ground contact was defined when the GRF signal changed at a rate of 10,000 N/s between successive time points [16]. From trimmed data, ground contact time (GCT; Figure 1) and rebound jump flight time (FT; Figure 1) were estimated using methods described previously [16].

#### 2.3.3. RSI Computations

RSI was first computed using the conventional approach, which requires inputting ground contact time (s) and flight time (s) into Equation (2).

(2) $$Reactive\;Strength\;Index=\frac{Rebound\;Jump\;Height}{Ground\;Contact\;Time}=\frac{4.905\times {\left(0.5\times Flight\;Time\right)}^{2}}{Ground\;Contact\;Time}$$

RSI was then computed using the mixed-methods approach described by Baca [10]. This approach requires an estimate of estimated impact velocity (*v_estimated_*) and integrated vertical GRF data (*v_GRF_*) to be inputted into Equation (3). As described by Baca [10], estimated impact velocity can be estimated using 2-dimensional videography and body segment parameters. In the present study, estimated impact velocity was estimated using sagittal plane video data and de Leva [11] body segment parameter tables. Integrated vertical GRF data (*v_GRF_*) were obtained by inputting time-series data (GRF) corresponding to GCT into Equation (4). Equation (4) computes *v_GRF_*, which is the change in vertical velocity of the WBCoM during the time period where participants’ feet were in contact with the force platform. The difference of *v_GRF_* and *v_estimated_* equates to upward velocity of the WBCoM at the time instant of rebound jump take-off (see Equation (5)).

(3) $$Reactive\;Strength\;Index=\frac{Rebound\;Jump\;Height}{Ground\;Contact\;Time}=\frac{\left(\frac{{\left(v_{GRF}-v_{estimated}\right)}^{2}}{19.62}\right)}{Ground\;Contact\;Time}$$

(4) $$v_{GRF}=\frac{\int GRF}{Body\;Mass}$$

(5) $$Rebound\;Jump\;Take-off\;Velocity=v_{GRF}-v_{estimated}$$

Prior to statistical analysis, normality of data was confirmed using the Shapiro–Wilk test. For each drop height, differences in estimated and theoretical impact velocities were assessed using a *t*-test. To account for inflation, a Bonferroni correction was applied to the alpha cut-off value (adjusted α = 0.0167).

To evaluate the statistical relationship between RSI computational approaches, a linear regression was performed on data from each drop height using RSI conventional as the response variable and RSI mixed-methods as the predictor variable. In addition, the statistical effects of RSI computational approach and drop height were assessed using a 2 (computation approach) × 3 (drop height) Analysis of Variance (ANOVA). Multiple one sample *t*-tests were performed to evaluate the statistical relationship between estimated and theoretical impact velocities. All statistical analyses were performed using IBM SPSS Statistics for Windows (Version 25, IBM Corporation, Armonk, NY, USA). All hypothesis tests were conducted using an alpha level of 0.05.

## 3. Results

### 3.1. Assumption #1

**Figure 2:**
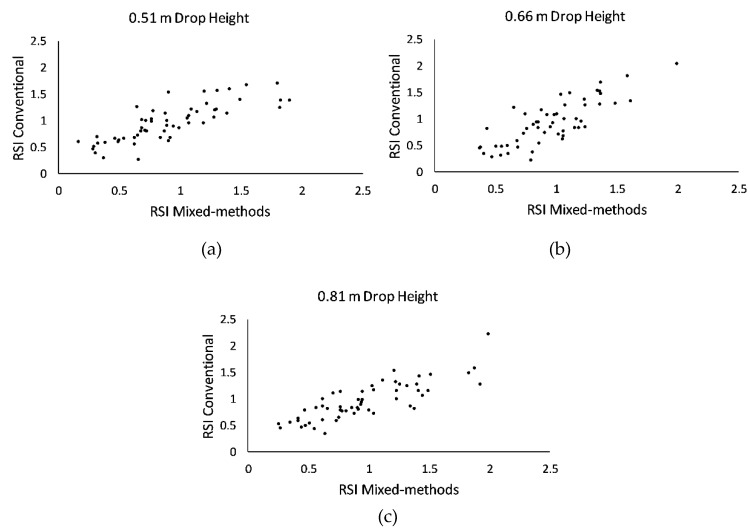
Scatter plot presentation of reactive strength index (RSI) data. (**a**) Data are averages from 70 participants that performed three trials of depth jumping at a drop height of 0.51 m; (**b**) Data are averages from 70 participants that performed three trials of depth jumping at a drop height of 0.66 m; (**c**) Data are averages from 70 participants that performed three trials of depth jumping at a drop height of 0.81 m.

RSI data are presented in Figure 2a–c. Linear regressions revealed a moderately strong association between RSI conventional and RSI mixed-methods (Table 1). Statistical associations were significant across all three drop height conditions (Table 1). 

Descriptive statistics for RSI data are presented in Table 2. Main effects were not observed for computational approach (*F* = 0.006; *p* = 0.938) or drop height (*F* = 0.157; *p* = 0.855). There was no significant interaction between computational approach and drop height (*F* = 0.765; *p* = 0.467).

### 3.2. Assumption #2

**Figure 3:**
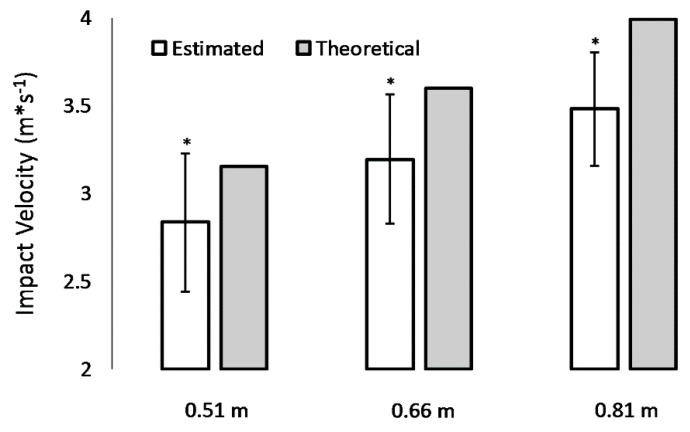
Graphical representation of mean comparisons. Dependent variables were theoretical and estimated impact velocity acquired from a sample of 70 young adults who performed a single trial of depth jumping at drop heights of 0.51, 0.66, and 0.81 m. *Estimated impact velocity was significantly lower versus theoretical (*p* < 0.05). Theoretical impact velocities were 3.16, 3.60, and 3.99 m*s^−1^ corresponding to the 0.51, 0.66, and 0.81 m drop heights, respectively.

Using an equation of constant acceleration (see Equation (1)), drop heights of 0.51, 0.66, and 0.81 m produce theoretical impact velocities of 3.16, 3.60, and 3.99 m*s^−1^ (Figure 3), respectively. Using 2-dimensional videography and de Leva [11] body segment parameters, mean estimated impact velocities were 2.84 ± 0.39 m*s^−1^ for the 0.51 m drop height condition (–10% versus theoretical), 3.20 ± 0.37 m*s^−1^ for the 0.66 m drop height condition (–11% versus theoretical), and 3.48 ± 0.32 m*s^−1^ for the 0.81 m drop height condition (–13% versus theoretical; Figure 3). Across all drop height conditions, estimated impact velocities were significantly lower versus theoretical impact velocities (*p* < 0.001; Figure 3).

## 4. Discussion

The aim of the present study was to evaluate the internal validity of the RSI. Conventionally, several assumptions are made when computing and interpreting the RSI. First, when estimating rebound jump height from flight time, it is assumed that the angular positioning of body segments is equivalent at the time instances of rebound jump take-off and rebound jump landing. Second, it is assumed that the vertical displacement of the WBCoM during the drop phase of depth jumping is mathematically equivalent to the height of the platform used to perform the drop. Results of this investigation suggest that these assumptions threaten the internal validity of conventional RSI computation and the interpretation of RSI scores, respectively. 

Estimated impact velocities were between 10% and 13% lower than theoretical expectations (Figure 3). This suggests that participants lowered their whole-body center of gravity prior to initiating the drop phase of the depth jump. Moreover, standard deviations for estimated impact velocities were between 0.32 and 0.39 m*s^−1^ (Figure 3). This indicates that the technique used to initiate the drop phase varies substantially across individuals. RSI scores are often interpreted within the context of drop height. While it is well known that impact velocities are not always related to drop height, results of the present study support taking a cautious approach when comparing scores across individuals. RSI scores are also used to aid in the optimization of plyometric intensity. Results suggest for practitioners to be mindful that estimated impact velocities are variable and may, on average, be lower than theoretical expectations.

Prior literature supports a mixed-methods approach for evaluating the linear mechanics of the WBCoM in depth jumping [10]. Results from Arnold Baca [10] confirm that substantial measurement error is introduced when using flight time to approximate rebound jump height in depth jumping. Specifically, Baca observed that the flight time method overestimates rebound jump height by as much as 8.4%. Moreover, the findings by Baca [10] suggest that assumptions made in conventional RSI computations are a threat to internal validity and that a mixed-methods approach using 2-dimensional videography, body segment parameters, and force platform data is preferred. In fact, Baca [10] observed that a mixed-methods approach, like that of the present investigation, estimates rebound jump height to an accuracy of 7 ± 13 mm when compared against a double force-platform reference.

In the present investigation, moderately strong linear associations (*R*^2^ = 0.685–0.741; Table 1) were observed between the conventional and mixed-methods RSI computation approaches. RSI conventional tended to overestimate RSI mixed-methods for scores under 1.00 and underestimate RSI mixed-methods for scores greater than 1.00 (Table 3). Results suggest for sport and clinical practitioners to take caution when making inferences about RSI conventional and consider the internal validity of scores prior to making recommendations to an athlete or client. 

Interestingly, the ANOVA failed to detect a main effect for computational approach and interaction between computational approach and drop height. It is probable that the wide dispersion of RSI scores above and below 1.00 (Figure 2) resulted in a bidirectional distribution of computational error (Table 3). Thus, while the lack of a significant main effect for computational approach supports acceptable agreement, it fails to account for the magnitude of measurement error between RSI conventional and RSI mixed-methods and should be discounted when considering the results of the study in entirety.

## 5. Conclusions

Results of the present study suggest that assumptions made in conventional RSI computation may introduce threats to internal validity. Researchers with access to motion capture and force platform technology may consider using a mixed-methods approach for computing the RSI, which likely maximizes the internal validity of scores acquired in laboratory settings. Results also suggest that sport and clinical practitioners should practice caution when interpreting RSI scores computed using the conventional approach. Additional research literature is needed to confirm the validity of the RSI and the reliability of the present study applied across additional samples, with increased focus on computational methods.

## Figures and Tables

**Table 1:** Regression model outputs. Predictor = RSI Mixed-Methods. Response = RSI Conventional.

Drop Height	*R* ^2^	*F*	*p-*Value	β	*p-*Value	Constant	*p-*Value
0.51 m	0.685	119.3	<0.001	0.648	<0.001	0.365	<0.001
0.66 m	0.692	123.3	<0.001	0.590	<0.001	0.406	<0.001
0.81 m	0.741	157.2	<0.001	0.644	<0.001	0.328	<0.001

**Table 2:** RSI descriptive statistics. Data are reported as mean ± standard deviation.

Drop Height	RSI Conventional	RSI Mixed-Methods
0.51 m	0.97 ± 0.37	0.94 ± 0.48
0.66 m	0.99 ± 0.37	1.00 ± 0.52
0.81 m	0.98 ± 0.38	1.00 ± 0.53

**Table 3:** Hypothetical scores for RSI conventional and mixed-methods based on regression results.

Drop Height	RSI Conventional	RSI Mixed-Methods
0.51 m	0.60	0.36
	1.00	0.98
	1.40	1.60
0.66 m	0.60	0.33
	1.00	1.01
	1.40	1.68
0.81 m	0.60	0.42
	1.00	1.04
	1.40	1.66
